# Where Do Bone-Targeted Agents RANK in Breast Cancer Treatment?

**DOI:** 10.3390/jcm2030089

**Published:** 2013-08-28

**Authors:** Roger von Moos, Ian Haynes

**Affiliations:** 1 Medical Oncology/Haematology, Kantonsspital Graubünden, Chur 7000, Switzerland; 2 Amgen Ltd., 1 Uxbridge Business Park, Uxbridge UB8 1DH, UK; E-Mail: ian.haynes@amgen.com

**Keywords:** RANK ligand, breast neoplasms, bone metastasis, bone metastasis-free survival, adjuvant therapy, skeletal-related event, bone-targeted agents, bisphosphonates, denosumab

## Abstract

Breast cancer cells preferentially metastasise to the skeleton, owing, in part, to the fertile environment provided by bone. Increased bone turnover releases growth factors that promote tumour cell growth. In turn, tumour cells release factors that stimulate further bone turnover, resulting in a vicious cycle of metastasis growth and bone destruction. The RANK-RANK ligand (RANKL) pathway plays a key role in this cycle, and inhibition of RANKL using the fully-human monoclonal antibody denosumab, has demonstrated efficacy in delaying skeletal complications associated with bone metastases in three phase 3 trials. Preclinical studies suggest that the RANKL pathway also plays a role in breast cancer tumourigenesis and migration to bone. In a subgroup analysis of the negative Adjuvant Zoledronic Acid to Reduce Recurrence (AZURE) trial, the bisphosphonate zoledronic acid showed potential for improving survival in patients who were postmenopausal; however, a prospective study in this patient population is required to validate this observation. Ongoing trials are examining whether adjuvant blockade of the RANKL pathway using denosumab can prevent disease recurrence in patients with high-risk breast cancer. These are building on analogous studies that have shown that denosumab improves bone metastasis-free survival in prostate cancer and suggested that it confers an overall survival benefit in non-small-cell lung cancer.

## 1. Introduction

Bone metastases are common in many types of solid tumours and occur in over 70% of individuals with advanced breast cancer [1]. They are associated with debilitating skeletal complications, commonly referred to as skeletal-related events (SREs) and comprising pathologic fracture, radiation to bone, surgery to bone or spinal cord compression [2]. As well as being associated with increased mortality [3,4], SREs can be associated with severe pain, impaired mobility and reduced quality of life [1,5]. Improvements in the management of breast cancer have led to increased survival times for patients with metastatic bone disease, but this means that the life-time risk of developing SREs has also increased.

The high frequency of bone metastasis in breast cancer can be attributed in part to patterns of venous circulation, with blood draining from the breast to the spinal veins [2]. The distribution of bone metastases reflects this network of blood flow. Thus, most common metastatic site is the axial skeleton, in particular the spine, sternum and ribs [2,6,7]. Although patterns of venous circulation explain the distribution of metastases to a degree, autopsy studies indicate that, if metastatic sites were solely determined by blood flow, there should be fewer bone metastases than are observed in breast cancer [8].

Tracking the migration and growth of tumour cells in a mouse model illustrated that the rate-limiting step in metastasis was not migration and extravasation into tissue, but was the ability of tumour cells to grow in the surrounding tissue [9]. This ability is determined by both the properties of the tumour cells themselves, and of the tissue they have migrated to. In 1889, Stephen Paget proposed the theory that bone provides an environment that is particularly conducive to breast cancer cell growth [10]. Extensive research has since demonstrated that it is the continuous turnover of bone that results in a fertile “soil” in which metastatic cells can “seed”. During bone resorption, the bone matrix releases a variety of growth factors that promote cell proliferation and survival [11,12]. The cells involved in resorption (osteoclasts) also produce angiogenic factors and matrix metalloproteinases that facilitate growth of new vasculature [13], an absolute requirement for tumour progression [8].

## 2. The RANK Pathway in Bone Metastasis

**Figure 1:**
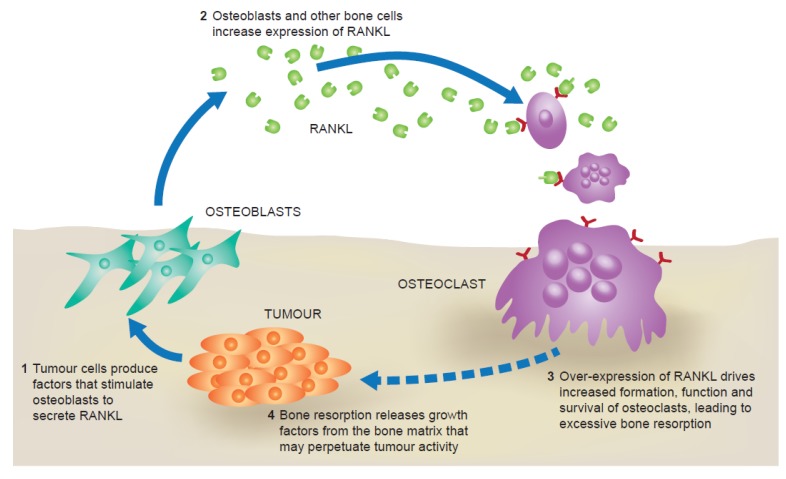
The “vicious cycle” of bone destruction in metastatic bone disease.

The favourable environment provided by bone is enriched further through interactions between tumour cells and some of the cells within the bone (osteoclasts and osteoblasts) [12]. These interactions stimulate bone resorption, thereby increasing the availability of tumour-promoting factors, which in turn results in tumour cell proliferation. If left unchecked, these reciprocal interactions result in a self-perpetuating cycle of bone destruction and tumour growth. The RANK ligand (RANKL)/RANK pathway is a key driver of this “vicious cycle” (Figure 1).

RANKL is a member of the tumour necrosis factor family of cytokines. When it binds to its receptor, RANK, expressed on osteoclast precursors, it promotes their differentiation, function and survival [14]. When breast cancer cells grow they produce several factors, such as parathyroid hormone-related peptide [15], that stimulate production of RANKL by stromal and osteoblast-lineage cells [16]. This in turn promotes osteoclastogenesis, increasing bone resorption and resulting in the release of more growth factors and angiogenic mediators that further stimulate tumour cell growth and proliferation [11,12,14]. Animal studies using mouse models of breast cancer metastasis to bone have demonstrated that administration of recombinant osteoprotegerin (OPG), the RANKL decoy receptor, decreases the number of tumour-associated osteoclasts and reduces levels of bone resorption, thus confirming a role for RANKL in tumour-induced, osteoclast-mediated bone destruction [17,18].

Denosumab, a fully-human monoclonal antibody that binds RANKL and neutralises its function, has demonstrated efficacy in reducing SREs in three phase 3 trials, including in patients with bone metastases associated with breast cancer, prostate cancer and other solid tumours or multiple myeloma [19,20,21,22], illustrating the part that the RANKL pathway plays in metastatic bone disease (Table 1). In these trials, denosumab was compared with the bisphosphonate zoledronic acid, the previous standard of care for patients with bone metastases. Denosumab significantly delayed the time to both first and subsequent SREs in patients with breast and prostate cancer compared with zoledronic acid, and was non-inferior in delaying SREs in patients with other solid tumours or multiple myeloma. Denosumab also significantly decreased levels of bone turnover in all three studies, as determined by suppression of two bone turnover markers: urine *N*-telopeptide corrected for creatinine (uNTx), and bone-specific alkaline phosphatase (BSAP) [19,20,21]. Pre-specified analysis of the results from the breast cancer trial found that denosumab significantly delayed progression to moderate or severe pain in patients with no or mild pain at baseline [23]. Individuals in the denosumab arm of the study were also more likely to have an improvement in their quality-of-life scores and less likely to have a decline in quality of life than those in the zoledronic acid arm [24].

**Table 1:** Results from a phase 3, randomised, double-blind study comparing denosumab with zoledronic acid for the prevention of skeletal-related events in patients with bone metastases associated with breast cancer [19].

Study arms	Zoledronic acid 4 mg i.v. Q4W	Denosumab 120 mg s.c. Q4W
Number of patients	1020	1026
Time to first SRE, months	26.4	NR
HR (95% CI)	0.82 (0.71–0.95)	
*p* Value	<0.001 (non-inferiority) 0.01 (superiority)	
Time to first and subsequent SRE, RR (95% CI)	0.77 (0.66–0.89)	
*p* Value	0.001 (superiority)	

*p* Values for superiority were adjusted for multiplicity; CI, confidence interval; HR, hazard ratio; i.v., intravenous; NR, not reached; Q4W, every 4 weeks; RR, rate ratio; s.c., subcutaneous; SRE, skeletal-related event.

## 3. RANKL and Tumour Progression

**Figure 2:**
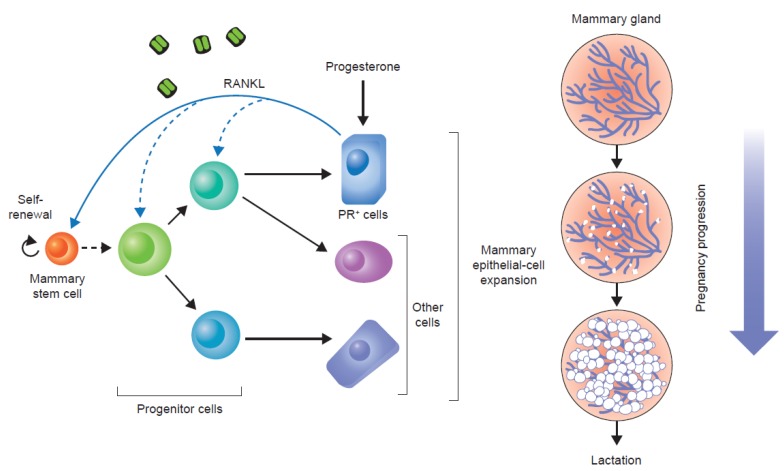
RANKL in mammary gland epithelial cell proliferation. Following the binding of progesterone to its receptor, RANKL is produced and acts in a paracrine fashion to stimulate mammary gland epithelial cell expansion. PR, progesterone receptor. Reprinted from [32].

Pre-clinical evidence suggests that the RANKL pathway not only functions in the establishment and growth of bone metastases, it also plays a role earlier in the breast cancer disease continuum [13]. RANKL and RANK are expressed in a number of cell types, including mammary gland epithelial cells [25]. While hormone-driven proliferation of mammary gland epithelial and stem cells can be partially explained by the autocrine effect that results from progesterone binding its receptor, the majority of proliferating cells are progesterone receptor-negative. This paracrine effect appears to be mediated by the RANKL pathway [26,27]. Moreover, murine studies have revealed a role for RANK and RANKL in hormone-driven mammary gland development during pregnancy (Figure 2) [28].

**Figure 3:**
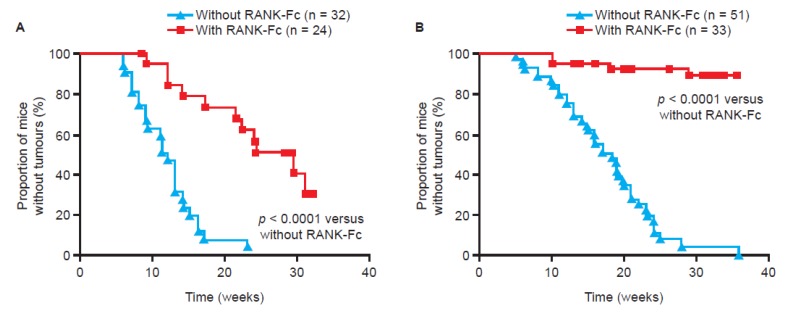
Blockade of RANK through pharmacological inhibition or genetic inactivation inhibits tumour formation in mice. Tumour growth following the administration of the carcinogen 7,12-dimethylbenzanthracene (DMBA) and the progesterone derivative medroxyprogesterone acetate (MPA), with and without concomitant treatment with the RANK inhibitor RANK-Fc, in (**A**) transgenic mice overexpressing RANK and (**B**) wild-type mice [29]. Reprinted from [29].

Notably, both RANK and RANKL are also expressed in tumour and stromal cells from human breast cancer [29,30]. Two key studies in mouse models have demonstrated a potential role for the RANKL pathway in mediating progesterone-driven breast cancer. One study used a transgenic model in which RANK was deleted from mammary gland epithelial cells [31]. The other study engineered overexpression of RANK in a mouse model, and used pharmacological inhibition (the RANKL antagonist, RANK-Fc) to block the pathway [29]. Hormonal stimulation (using a synthetic progesterone derivative, medroxyprogesterone acetate (MPA)) markedly increased levels of RANKL in both the transgenic mice overexpressing RANK and the wild-type mice, and triggered epithelial cell proliferation [29,31]. Mice over-expressing RANK had a much higher incidence of mammary tumours following co-administration of MPA and a carcinogen (7,12-dimethylbenzanthracene (DMBA)) than wild-type mice. Blocking the pathway using RANK-Fc dramatically decreased the incidence of tumour formation in both types of mice (Figure 3) [29]. Furthermore, comparing mammary cell proliferation following RANK-Fc inhibition with proliferation following inhibition of the progesterone receptor found that the RANKL pathway was responsible for the majority of the proliferatory effect [29]. Therefore, similar to its role in mammary gland development (Figure 2), the RANKL pathway appears to be a key mediator of progesterone-driven cell proliferation in tumourigenesis.

**Figure 4:**
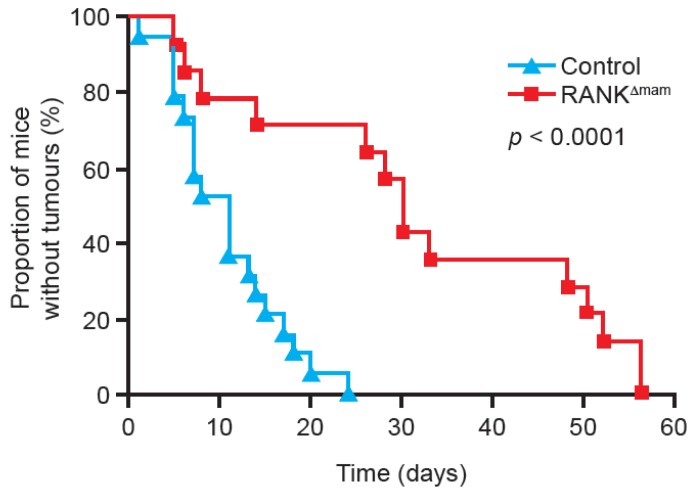
RANK knock-out from mammary gland epithelia inhibits tumour formation. Tumour growth following the administration of the carcinogen DMBA and the progesterone derivative MPA in mice with inactivated mammary gland epithelial cell RANK expression (RANK^Δmam^) and in wild-type mice (control) [31]. Reprinted from [31].

In contrast to the effects seen with overexpression of RANK, mice with mammary gland epithelial cell RANK gene deletion had decreased cell proliferation upon progesterone stimulation compared with wild-type mice. They also exhibited a marked delay in tumour formation and increased overall survival when stimulated with MPA and DMBA (Figure 4) [31]. The protective effect of RANK deletion occurred only if it was deleted from mammary gland epithelia: Deleting RANK from other cell types did not reduce mammary tumour formation. This pattern suggests an additional, cell-specific role of the RANKL pathway that is restricted to mammary gland epithelial cells. Furthermore, administration of zoledronic acid, which has been demonstrated to inhibit the functioning of osteoclasts through the mevalonate pathway by blocking post-translational modification of proteins necessary for their survival [33], had no effect on mammary tumour growth. This again suggests that the RANKL pathway involvement in mammary tumourigenesis is independent of its role in bone physiology [29].

Results from *in vitro* and *in vivo* studies suggest that RANKL may also be involved in tumour cell migration to the skeleton. *In vitro* stimulation of breast cancer cells with RANKL resulted in actin polymerisation and cell migration, both pre-requisites for metastasis [30]. A mouse model of melanoma (which frequently metastasises to bone) demonstrated that treatment with a recombinant form of the RANKL decoy receptor, OPG, reduced tumour burden in bone and prevented development of paralysis [30]. Interestingly, treatment with zoledronic acid did not reduce tumour burden in bone [30], suggesting that RANKL mediates its chemotactic function through a mechanism independent of its effects on osteoclasts.

In support of these roles of the RANKL pathway in tumour progression, analysis of RANK and OPG gene expression in patients with breast cancer found that low levels of RANK and high levels of OPG correlated with improved disease-free survival and overall survival, and that RANK expression positively correlated with the risk of developing bone metastases [34]. A study of RANK overexpression in breast cancer and mammary epithelial cells found that RANK induces de-differentiation of cells and epithelial-mesenchymal transition; changes that are associated with tumourigenesis, and invasion and metastasis, respectively [35].

## 4. Anti-Tumour Effects of Bone-Targeted Agents in Clinical Trials

The potential anti-tumour effects of bone-targeted agents have been explored in several trials. Five large, open-label clinical trials, Austrian Breast and Colorectal Cancer Study Group (ABCSG-12), AZURE, immediate *versus* delayed administration of zoledronic acid in patients with early breast cancer receiving adjuvant letrozole (E-ZO-FAST), Zometa-Femara Adjuvant Synergy Trial (Z-FAST) and (ZO-FAST), have investigated the adjuvant use of zoledronic acid in patients with breast cancer, with mixed results (Table 2) [36,37,38,39]. In ABCSG-12, premenopausal patients receiving goserelin with tamoxifen or anastrozole were randomly assigned to either receive zoledronic acid 4 mg every 6 months, or to have no anti-resorptive therapy. Postmenopausal patients enrolled in E-ZO-FAST, Z-FAST and ZO-FAST were randomly assigned to either receive zoledronic acid upfront (4 mg every 6 months), or to have treatment with zoledronic acid delayed until fracture or a decrease in bone mineral density (BMD) [38]. In AZURE patients received standard therapy with or without zoledronic acid. The bisphosphonate was administered at 4 mg every 3–4 weeks for the first six doses, then every 3–6 months thereafter [37].

Improvements in disease-free survival were achieved in the ABCSG-12 and ZO-FAST studies, and an improvement in overall survival was observed in the ABCSG-12 study [36,39]. Further analysis demonstrated, however, that in the ABCSG-12 study, this benefit was confined to patients aged over 40 years [36]. Exploratory analyses of the ZO-FAST results revealed a potential survival advantage for patients who were either over 60 years old or who had been postmenopausal for over 5 years (HR = 0.50; *p* = 0.022) [39]. Despite having the same study design as ZO-FAST, no improvements in disease-free survival were seen in either E-ZO-FAST or Z-FAST [38]. The recently updated Cochrane analysis by Wong *et al*. [40] further reviewed these data along with the outcomes from similar studies conducted with oral clodronate. Based on the results of their meta-analysis, the authors concluded that overall, bisphosphonates do not prevent overall disease recurrence (RR 0.97; 95% CI 0.81, 1.16; *p* = 0.75) or improve survival (RR 0.84; 95% CI 0.68, 1.04; *p* = 0.11) in early breast cancer when compared with no bisphosphonates. When compared with delayed bisphosphonate treatment, early bisphosphonate treatment did not prevent overall recurrence (RR 0.85; 95% CI 0.49, 1.50; *p* = 0.58) and was even associated with a trend towards reduced survival (RR 1.45; 95% CI 0.44, 4.70; *p* = 0.54).

**Table 2:** Results of five large, open-label clinical trials investigating the adjuvant use of zoledronic acid in patients with breast cancer.

Study	ABCSG-12 [36]	AZURE [37]	E-ZO-FAST [38]	Z-FAST [38]	ZO-FAST [39]
Study design	Goserelin with tamoxifen or anastrozole ± zoledronic acid (4 mg Q6M)	SOC ± zoledronic acid (6 doses 4 mg Q3W–Q4W, then 4 mg Q3M–Q6M)	Upfront zoledronic acid (4 mg Q6M) *vs*. delayed treatment	Upfront zoledronic acid (4 mg Q6M) *vs*. delayed treatment	Upfront zoledronic acid (4 mg Q6M) *vs*. delayed treatment
Number of patients	1803	3360	527	602	1065
Primary endpoint	Disease-free survival	Disease-free survival	LS BMD at 12 months	LS BMD at 12 months	LS BMD at 12 months
Median length of follow up, months	76	59	36	54	60
Disease-free survival, HR (95% CI)	0.73 (NR)	0.98 (0.85, 1.13)	1.76 (0.83, 3.69)	0.80 (0.45, 1.41)	0.66 (0.44, 0.97)
*p* Value	0.022	0.79	NS	NS	0.0375
Overall survival, HR (95% CI)	0.59 (NR)	0.85 (0.72, 1.01)	NR	NR	0.69 (0.42, 1.14)
*p* Value	0.027	0.07	NR	NR	0.1463

BMD, bone mineral density; CI, confidence interval; HR, hazard ratio; LS, lumbar spine; NR, not reported; NS, not significant; SOC, standard of care; *vs*., *versus*.

AZURE included a heterogeneous group of both premenopausal and postmenopausal patients. In this study, which was discontinued by the data management committee on the grounds of futility, no improvements in disease-free survival or overall survival were seen in the overall study population [37]. However, in an unadjusted sub-group analysis of patients who had been postmenopausal for more than 5 years, improvements in both disease-free survival (HR = 0.75; *p* = 0.02) and overall survival (HR = 0.74; *p* = 0.04) were observed. No benefit was seen in any of the other patient subsets (premenopausal, perimenopausal or unknown menopausal status), and there was an adverse effect of zoledronic acid treatment on non-bone invasive disease-free survival in these patients (HR = 1.32). With respect to these findings, Wong *et al*. [40] noted that combining the data for the post-menopausal subgroups from the AZURE study and the artificially-induced menopausal women in the ABCSG-12 study had a significant relative risk reduction in overall recurrence *versus* control (RR 0.71; 95% CI 0.59, 0.85; no statistical heterogeneity *p* = 0.46). However, they cautioned against over interpretation given that oestrogen levels were not measured in these studies and thus such sensitivity analyses are only speculative.

The apparent differences in treatment effect according to hormone status are not specific to zoledronic acid: A recent study suggested the bisphosphonate clodronate, which acts via a different pathway to zoledronic acid, may also improve disease-free survival in older patients with breast cancer [41]. It should be note, however, that another study of adjuvant clodronate in patients with primary breast cancer found it was associated with significantly worse overall survival, compared with a control group, although this study did include both premenopausal and postmenopausal patients [42]. The reasons for this possible hormone-specific effect on survival are not fully understood. Some hypotheses suggest oestrogen provides a protective effect on bone that reduces the potential for skeletal metastases, thereby reducing the potential benefit bone-targeted agents would provide. Oestrogen is known to prevent bone turnover; premenopausal women may therefore be less likely to benefit from agents that suppress bone resorption than postmenopausal women. Hormones also influence the cytokine milieu in the bone microenvironment, and so the environment in which the metastatic cells “seed” could potentially be quite different depending on the hormone status of patients [43]. Alternatively, oestrogen could be mediating a pro-tumour effect that counteracts the anti-tumour effect of bisphosphonates. For example, oestrogen can polarise the immune system to a tumour-permissive state by supporting M2 (tumour-promoting) macrophages and increasing cell resistance to cytotoxic natural killer cells [44]. Furthermore, some cytokines, such as transforming growth factor-beta (TGF)-β, are regulated by both hormonal pathways and by the mevalonate pathway, suggesting zoledronic acid and oestrogen may act antagonistically [43]. However, as clodronate does not act via the mevalonate pathway [33], this mechanism is unlikely to mediate the differences in treatment effect for this drug. Establishing whether hormone-dependent treatment effects are specific to all drugs that inhibit bone turnover, or whether the effects are bisphosphonate-specific, may help elucidate the specific pathways involved and improve patient selection. In addition to differences in hormone profile between younger and older women with breast cancer, a recent study examining gene expression found young women with breast cancer are more likely to have high RANKL expression than older women. Therefore, in pre-menopausal women with breast cancer, who are unlikely to benefit from adjuvant bisphosphonates, the RANKL pathway may be a potential therapeutic target [45].

The preclinical evidence discussed earlier suggests that denosumab may have a role in preventing disease progression in breast cancer. Furthermore, clinical data from several studies support the pre-clinical evidence suggesting a role for RANKL in tumour progression in prostate cancer. In a phase 3 study of patients with high-risk, non-metastatic, castration-resistant prostate cancer, RANKL blockade using denosumab significantly improved bone metastasis-free survival by 4.2 months compared with placebo (HR = 0.85; *p* = 0.028), illustrating how manipulation of the RANKL pathway may affect disease progression [46]. A separate analysis of patients undergoing radical prostatectomy found that increased serum concentration of RANKL is an independent prognostic risk factor for biochemical disease recurrence [47]. In addition, a sub-group analysis of clinical data from the phase 3 trial that compared denosumab with zoledronic acid in patients with solid tumours or multiple myeloma found that treatment with denosumab was associated with improved overall survival *versus* treatment with zoledronic acid in patients with non-small cell lung cancer (NSCLC) (HR = 0.78; *p* = 0.01) [48]. These outcomes may be a RANKL pathway-specific effect: Results from the Zometa European Study (ZEUS), which compared standard therapy with and without zoledronic acid treatment in patients with high risk prostate cancer, suggest that zoledronic acid treatment does not improve either overall survival or the incidence of bone metastasis [49]. Furthermore, zoledronic acid does not appear to prolong overall or disease-free survival in patients with NSCLC [50].

Building on the strong preclinical rationale and analogous studies in other tumour types, the ongoing D-CARE (denosumab as adjuvant treatment for women with high risk early breast cancer receiving neoadjuvant or adjuvant therapy) study is investigating whether adjuvant denosumab (120 mg subcutaneously monthly for the first 6 months and every 3 months thereafter) can prevent disease recurrence in patients with high-risk breast cancer [51,52]. This international, randomised, double-blind phase 3 study is evaluating bone metastasis-free survival, disease-free survival and overall survival in approximately 4500 women with stage II or III breast cancer at high risk of recurrence. The trial has recently completed recruitment of patients. The results of the ABCSG-18 trial will also be of interest; this trial is primarily investigating using a lower dose of denosumab (60 mg every 6 months) to reduce the rate of clinical fracture in patients with non-metastatic breast cancer, but will also report bone metastasis-free, disease-free and overall survival as secondary endpoints [53].

## 5. Conclusions

There is little doubt that both denosumab and bisphosphonates can delay the occurrence of SREs in patients with bone metastases, thereby reducing pain and improving patients’ quality of life. However, the role of bone-targeted agents in the adjuvant setting is unclear. Data suggest that bisphosphonates may be beneficial in certain subsets of patients with breast cancer, but may cause harm in others. Further studies with sufficient power and prospectively defined endpoints are required to confirm the population that should be targeted and to determine the risk/benefit profile for treatment. Evidence from other tumour types indicates a potential role for denosumab in delaying progression to bone metastasis and possibly in improving overall survival. There is also a strong preclinical rationale for blocking the RANKL pathway at an earlier stage of breast cancer treatment to delay disease progression. Ongoing clinical trials will determine whether manipulating the RANKL pathway at an earlier stage in breast cancer will be a valuable therapeutic strategy.
